# Galactose-Deficient IgA1 as a Candidate Urinary Polypeptide Marker of IgA Nephropathy?

**DOI:** 10.1155/2016/7806438

**Published:** 2016-08-28

**Authors:** Hitoshi Suzuki, Landino Allegri, Yusuke Suzuki, Stacy Hall, Zina Moldoveanu, Robert J. Wyatt, Jan Novak, Bruce A. Julian

**Affiliations:** ^1^Division of Nephrology, Juntendo University Faculty of Medicine, Tokyo 113-8421, Japan; ^2^University of Alabama at Birmingham, Birmingham, AL 35294, USA; ^3^University of Parma, 43100 Parma, Italy; ^4^University of Tennessee Health Sciences Center, Memphis, TN 38103, USA

## Abstract

In patients with IgA nephropathy (IgAN), circulatory IgA1 and IgA1 in mesangial deposits contain elevated amounts of galactose-deficient IgA1 (Gd-IgA1). We hypothesized that a fraction of Gd-IgA1 from the glomerular deposits and/or circulation may be excreted into the urine and thus represent a disease-specific biomarker. Levels of urinary IgA and Gd-IgA1 were determined in 207 patients with IgAN, 205 patients with other renal diseases, and 57 healthy controls, recruited in USA, Japan, and Italy. Urinary IgA was similarly elevated in patients with IgAN and renal-disease controls compared with healthy controls. However, urinary Gd-IgA1 levels were higher in patients with IgAN (IgAN, 28.0 ± 17.9; disease controls, 20.6 ± 17.4 units/mg urinary creatinine; *P* < 0.0001). Lectin western blotting data confirmed these results. In IgAN patients, levels of urinary Gd-IgA1 correlated with proteinuria (*P* < 0.001). When we purified IgA from serum and urine of an IgAN patient, the relative proportion of Gd-IgA1 to total IgA1 was higher in the urine compared with serum, suggesting selective excretion of Gd-IgA1 in IgAN. In summary, urinary excretion of Gd-IgA1 was elevated in patients with IgAN and the urinary Gd-IgA1 levels correlated with proteinuria. Urinary Gd-IgA1 may thus represent a disease-specific biomarker of IgAN.

## 1. Introduction

IgA nephropathy (IgAN) is the most common primary glomerulonephritis worldwide. Most cases of IgAN are discovered incidentally by abnormal urinalysis (hematuria/proteinuria) [1]. Diagnosis of IgAN is possible only by examination of cortical renal tissue obtained by biopsy, a procedure with inherent risks. As a consequence, the diagnosis is frequently delayed until the late clinical stages of disease, often beyond the time-point at which therapeutic intervention may be successful.

Galactose-deficient IgA1 (Gd-IgA1) has been identified as one of the key effector molecules in pathogenesis of IgAN, although the underlying molecular mechanisms are still under investigation [2–4]. A multihit hypothesis regarding pathogenesis of IgAN has been proposed [5]. In this hypothesis, four major steps are required for onset and progression of IgAN: overproduction of Gd-IgA1, generation of autoantibodies specific for the galactose-deficient glycans of Gd-IgA1, formation of immune complexes, and deposition of those complexes in glomeruli [5]. Gd-IgA1 has a crucial role in the pathogenesis of IgAN, with the outcomes likely modulated by contributing genetic factors and secondary immune dysregulation [6, 7]. The serum levels of Gd-IgA1 are associated with disease progression [8] and the abnormal glycosylation of IgA1 is a key determinant of glomerular affinity [9]. In fact, two studies revealed that glomerular IgA1 in patients with IgAN is enriched for the aberrantly glycosylated forms [10, 11]. Kinetics of glomerular deposition of human IgA1 in mice indicated that continuous clearance mechanisms countering IgA deposition are present in the glomerulus [12]. These mechanisms likely include proteolytic degradation of IgA1 taken up by mesangial cells [13]. Thus, glomerular IgA deposits in IgAN may be explained as an imbalance between deposition and clearance [12]. We hypothesized that a fraction of Gd-IgA1 from the glomerular deposits is excreted into the urine and thus represents a disease-specific biomarker of IgAN.

Establishment of a noninvasive diagnostic tool would be an important advance in the management of patients with IgAN worldwide. This study aims to assess the utility of lectin ELISA using* Helix aspersa* agglutinin (HAA, a GalNAc-specific lectin) for detection of urinary Gd-IgA1, with the goal to differentiate patients with IgAN from healthy controls and patients with other forms of renal disease.

## 2. Materials and Methods

### 2.1. Urine Samples

Spot urine samples were collected from 207 patients with biopsy-proven IgAN (59 from USA, 97 from Japan, and 51 from Italy), and 57 healthy controls (31 from USA and 26 from Japan) (Table 1). The renal-disease control urine samples were collected from 205 patients with other renal diseases (69 from USA, 25 from Japan, and 111 from Italy; Table 1), including lupus nephritis, focal segmental glomerulosclerosis, membranous nephropathy, diabetic nephropathy, minimal change disease with nephrotic syndrome, and polycystic kidney disease. All urine samples from IgAN patients and renal-disease controls were collected just before renal biopsy. Quantitative proteinuria was measured in each hospital. Hematuria was categorized as absent, 1+, 2+, or 3+ based on urinary test strip. The study was approved by the Institutional Review Boards in each institution.

### 2.2. ELISA Determination of IgA

IgA levels in urine samples were determined by capture ELISA. For coating ELISA plates, F(ab′)_2_ fragments of goat IgG specific for human IgA (*α* chain-specific) (Jackson ImmunoResearch Labs, West Grove, PA) were used and developed with biotin-labeled goat F(ab′)_2_ of IgG antibody against human IgA (Biosource, Camarillo, CA).

### 2.3. Determination of Gd-IgA1

F(ab′)_2_ fragment of goat IgG specific for human IgA (Jackson ImmunoResearch Labs, West Grove, PA) was coated onto ELISA panels (3 *μ*g/mL). Serially diluted samples were applied on the plates and the captured IgA was treated with 10 mU/mL neuraminidase (NA; Roche Diagnostic Corp., Indianapolis, IN) to remove terminal sialic acid residues. After washing, the samples were reacted with biotin-labeled GalNAc-specific lectin from* Helix aspersa* (Sigma, St. Louis, MO) followed by HRP-avidin and peroxidase substrate. Absorbance was measured at 490 nm. The results were calculated relative to HAA reactivity of a standard Gd-IgA1 (Ale) myeloma protein (its relative HAA reactivity was set to 100%).

### 2.4. Western Blotting

Urine samples were analyzed under nonreducing conditions by SDS-PAGE and blotted on PVDF membrane and probed with IgA heavy chain-specific antibody or HAA lectin. The blots were developed and visualized using enhanced chemiluminescence. Amount of the samples loaded was normalized to urinary creatinine.

### 2.5. Statistical Analysis

Data are expressed as means ± SEM. Comparison of groups was performed using univariate ANOVA;* post hoc* Bonferroni correction was used for multiple comparisons. Correlation between two groups was performed by regression analysis. *P* < 0.05 was considered significant. These statistical analyses were performed using the Prism software (GraphPad Software Inc., La Jolla, CA).

## 3. Results

### 3.1. Clinical Urinary Studies

**Figure 1:**
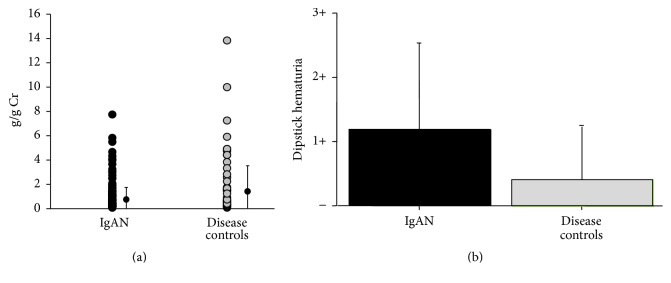
Clinical urinalysis for patients with IgAN and other renal diseases. (a) Urinary protein (UP) per urinary creatinine (UCr) ratio (g/g) was lower in patients with IgAN than in renal-disease controls (*P* < 0.05). (b) Degree of dipstick hematuria was higher in patients with IgAN than in renal-disease controls (*P* < 0.05).

Urinary protein per creatinine (Cr) ratio was lower in patients with IgAN than in renal-disease controls (IgAN, 0.9 ± 0.9; disease controls, 1.5 ± 3.0; g/gCr; *P* < 0.05) (Figure 1(a)). However, the degree of hematuria was higher in patients with IgAN than in the renal-disease controls (*P* < 0.05) (Figure 1(b)).

### 3.2. Urinary IgA and Gd-IgA1

**Figure 2:**
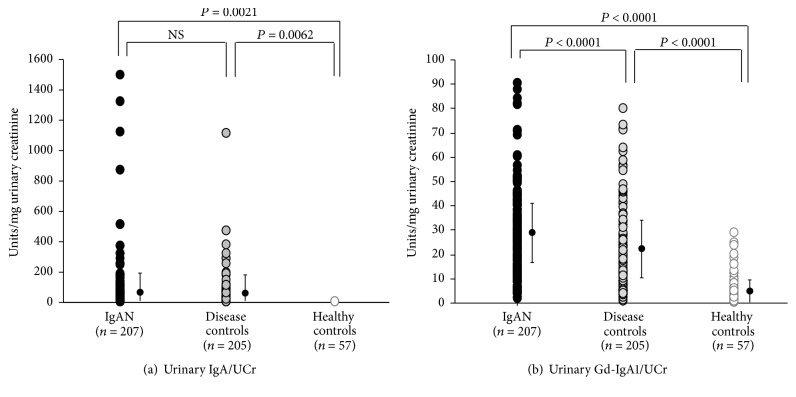
Urinary IgA was increased in IgAN patients and the renal-disease control patients compared to that in healthy controls, but urinary Gd-IgA1 excretion was higher in patients with IgAN than in renal-disease controls. (a) Urinary IgA was elevated in patients with IgAN as well as in disease controls. (b) Urinary Gd-IgA1 excretion was greater in patients with IgAN than in renal-disease controls (*P* < 0.0001).

The amount of urinary IgA was higher in both groups of patients with renal disease compared to that in healthy controls (Figure 2(a)). However, patients with IgAN excreted greater amounts of Gd-IgA1 than did the renal-disease or healthy controls (IgAN, 28.0 ± 17.9; renal-disease controls, 20.6 ± 17.4; healthy controls, 6.6 ± 6.7 units/mg urinary creatinine; *P* < 0.0001 for IgAN patients* versus* renal-disease controls and IgAN patients* versus* healthy controls, *P* < 0.0001 for renal-disease controls* versus* healthy controls) (Figure 2(b)).

### 3.3. HAA-Lectin Western Blotting Confirmed Increased Levels of Urinary Gd-IgA1

**Figure 3:**
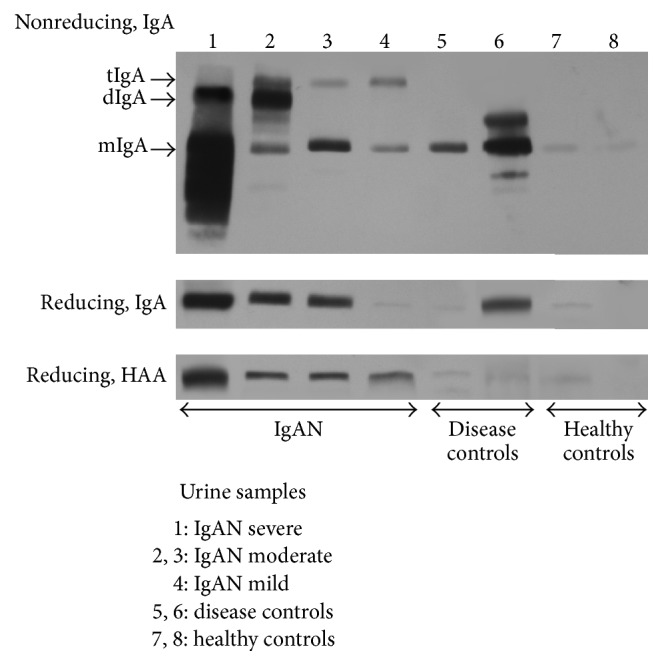
HAA-lectin western blotting confirmed increased urinary excretion of Gd-IgA1 in IgAN patients. Urine samples were normalized to urinary creatinine concentration. IgA western blotting under reducing and nonreducing conditions using urine samples from four patients with IgAN (lanes 1 to 4), two patients with lupus nephritis (lanes 5 and 6), and two healthy controls (lanes 7 and 8). Depending on the severity of proteinuria, varying amounts of IgA were excreted in urine. Notably, all four patients with IgAN, but none of the disease and healthy controls, had polymeric IgA in the urine samples. HAA-lectin western blotting under reducing conditions indicated that all patients with IgAN had urinary HAA-reactive IgA regardless of the amounts of urinary IgA (lane 1: IgAN with severe proteinuria (UP > 1.0 g/gCr), lanes 2 and 3: IgAN with moderate proteinuria (1.0 ≥ UP > 0.5 g/gCr), lane 4: IgAN with mild proteinuria (UP ≤ 0.5 g/gCr), lanes 5 and 6: lupus nephritis (UP > 1.0 g/gCr), and lanes 7 and 8: healthy controls).

We performed SDS-PAGE under reducing and nonreducing conditions, followed by western blotting for IgA, using urine samples from four patients with IgAN, two patients with lupus nephritis, and two healthy controls. Depending on the severity of proteinuria, the amounts of excreted IgA varied (Figure 3). Notably, all urine samples from the four tested patients with IgAN, but none of the samples from disease and healthy controls, had polymeric IgA (detected using nonreducing SDS-PAGE western blot). Next, we performed HAA-lectin western blotting after SDS-PAGE separation of samples under reducing conditions. All four samples from patients with IgAN showed HAA-reactive IgA, regardless of the amounts of urinary IgA. In contrast, urinary IgA from patients with lupus nephritis did not react with HAA, indicating that it did not include appreciable amounts of Gd-IgA1 (Figure 3).

### 3.4. Levels of Urinary Gd-IgA1 Correlated with the Degree of Proteinuria

**Figure 4:**
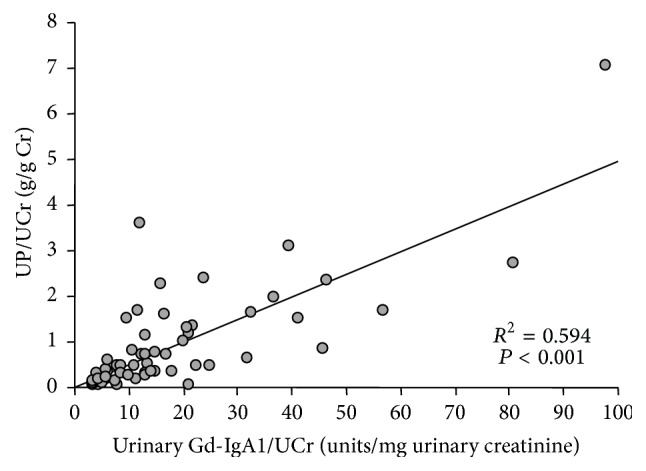
Amount of urinary Gd-IgA1 correlated with proteinuria (*P* < 0.001).

We analyzed possible association between the levels of urinary Gd-IgA1 and clinical findings in patients with IgAN. Levels of urinary HAA-reactive IgA1 correlated with proteinuria, measured as urinary protein per g creatinine (*R* ^2^ = 0.594, *P* < 0.001) (Figure 4). In renal-disease control subjects, the correlation coefficient between the levels of urinary HAA-reactive IgA1 and proteinuria was low compared to that in patients with IgAN (*R* ^2^ = 0.180, *P* = 0.033) (Supplemental Figure 1, in Supplementary Material available online at http://dx.doi.org/10.1155/2016/7806438). There was no correlation between urinary Gd-IgA1 levels and hematuria or episodes of macroscopic hematuria.

### 3.5. Urinary IgA1 in IgAN Exhibits a Higher Degree of Galactose Deficiency than Serum IgA1

**Figure 5:**
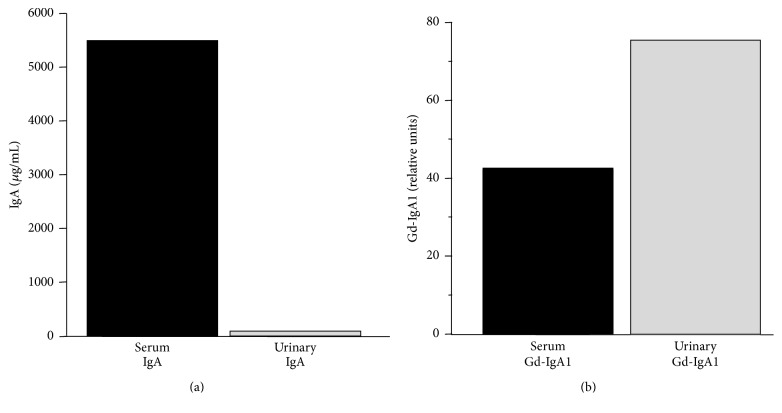
Urinary IgA1 exhibits higher degree of galactose deficiency compared with that of serum IgA1. (a) IgA levels in the serum and in the urine. (b) Relative proportion of Gd-IgA1 to total IgA1 was higher in the IgA1 purified from urine compared to that purified from serum of the same individual.

We isolated IgA from serum and urine of an IgAN patient. As expected, IgA concentration was much higher in the serum than that in the urine (Figure 5(a)). However, the relative degree of galactose deficiency was higher in IgA1 from the urine than from the serum, suggesting selective excretion of Gd-IgA1 (Figure 5(b)).

## 4. Discussion

IgAN is frequently associated with a poor prognosis, resulting in end-stage kidney disease in approximately 40% of cases within 20 years of the biopsy-proven diagnosis [1, 14]. Because the adverse outcome is partly a result of delayed diagnosis, strategies for early diagnosis leading to timely medical intervention are urgently needed. Urinalysis is often used to assess disease activity, although it has limitations. To this point, in the present study, proteinuria was present in 87% IgAN patients and 88% renal-disease controls. Unfortunately for patients with IgAN and proteinuria, there is no useful marker to distinguish patients with acute glomerular inflammatory lesions that may be amenable to therapy, such as cellular crescents, from those with scarred glomeruli with little inflammation. Therefore, the rationale for treatment based on magnitude of proteinuria in many clinical guidelines must be rigorously limited.

Gd-IgA1 has a crucial role in the pathogenesis of IgAN [15]. Serum levels of Gd-IgA1 are usually increased in patients with IgAN [3, 16], although this finding does not account for the development of the disease. First-degree relatives of patients with IgAN often have increased serum Gd-IgA1 levels, without clinical evidence of kidney disease [17]. Patients with IgAN have autoantibodies (IgG or IgA isotype) that recognize the galactose-deficient glycans of the hinge region of Gd-IgA1 to form immune complexes, either within the circulation [6] or after deposition of Gd-IgA1 in the glomerular mesangium [18, 19]. We have reported that IgG autoantibodies that recognize glycan-containing epitopes on Gd-IgA1 exhibit unique features in the complementarity-determining region 3 of the variable region of their heavy chains [20], apparently as a result of somatic mutation [21]. Furthermore, serum levels of IgG autoantibodies specific for Gd-IgA1 correlated with disease severity, as assessed by magnitude of proteinuria [20], histological prognostic group [16], and renal survival [22]. Thus, the serum level of anti-Gd-IgA1 IgG has shown potential as a biomarker for the clinical severity of IgAN. Also, in patients with IgAN the serum level of Gd-IgA1 is associated with the disease activity, manifested as hematuria or proteinuria [8, 23] as well as development of end-stage renal disease [22]. However, these serum biomarkers have not yet proven to identify patients with IgAN well enough to forego a kidney biopsy and are not sufficiently accurate in the assessment of disease activity. Thus, the search for better disease-specific biomarkers for IgAN continues.

Based on the mechanisms of disease for IgAN discussed above, we postulated that urinary excretion of IgA or Gd-IgA1 would distinguish patients with IgAN from patients with other forms of kidney disease or associate with disease expression. Excretion of IgA in patients with IgAN and renal-disease controls did not differ, but in both groups the amount was higher than the negligible excretion in healthy controls. This finding is in agreement with an earlier study that showed the fraction of proteinuria comprised by IgA did not differ between 29 patients with IgAN and 27 patients with proteinuria due to non-IgAN renal diseases [24]. Notably, all four patients with IgAN, but none of the renal-disease and healthy controls, had polymeric IgA in the urine (Figure 3). Furthermore, the results for Gd-IgA1 were more promising. We found greater urinary excretion of Gd-IgA1 in patients with IgAN than in the renal-disease controls. This result again supports the data [24] that indicated 68% of the IgAN patients excreted Gd-IgA1 whereas none of the renal-disease controls did. Moreover, this result is in agreement with our earlier observation that the serum levels of IgG autoantibodies correlated with levels of IgA-IgG immune complexes excreted in the urine [20].

The proportion of Gd-IgA1 to total IgA1 was higher in the urine compared with that in serum in the one patient with IgAN that we tested. This difference may be due to selective deposition of Gd-IgA1 in the glomerular mesangium. Aberrant glycosylation of IgA1 is a key determinant of glomerular affinity [9]. Other investigators have shown that glomerular IgA in patients with IgAN is aberrantly glycosylated IgA1 [10, 11]. There is a continuous process to remove IgA deposits in the glomerulus [12], as clinically illustrated by kidney transplantation. IgA deposits in an allograft from a patient with subclinical IgAN resolved within several weeks after engraftment into a patient with non-IgAN end-stage renal disease [25]; acute rejection may accelerate the process [26, 27]. Thus, glomerular IgA deposits in IgAN may result from an imbalance between deposition and clearance, a dynamic process that may be influenced by circulating loads of Gd-IgA1, availability of antibody specific for Gd-IgA1, or intensity of inflammatory activity in the mesangium. Measurement of urinary Gd-IgA1 may provide a snapshot as to the current status of glomerular injury in IgAN. We found that urinary Gd-IgA1 levels correlated with proteinuria.

In summary, urinary excretion of Gd-IgA1 discriminated patients with IgAN from patients with other proteinuric renal diseases. Furthermore, the level of urinary Gd-IgA1 correlated with proteinuria in patients with IgAN. Urinary Gd-IgA1 thus may represent a disease-specific biomarker of IgAN. These findings should be evaluated in a prospective study with contemporaneous renal biopsy and longitudinal urinary testing. It may be feasible to develop the assay into a novel noninvasive test to detect renal injury at early stages of IgAN and to monitor clinical manifestations and response to therapy.

## Supplementary Material

Supplemental Figure 1. In renal-disease-control subjects, the correlation coefficient between the levels of urinary Gd-IgA1 and proteinuria was low (R2=0.180, P=0.033) compared to that in patients with IgAN (see Figure 4 for comparison).

## Figures and Tables

**Table 1:** Study subjects.

	USA	Japan	Italy	Total
IgAN	59	97	51	207
Renal-disease controls	69	25	111	205
Healthy controls	31	26	0	57

Total	159	148	162	469
