# Supplementary figures and images for: Galactose-Deficient IgA1 as a Candidate Urinary Polypeptide Marker of IgA Nephropathy?
Source: Dis Markers. 2016 Aug 28;2016:7806438. doi: 10.1155/2016/7806438 (PMC5018335; doi:10.1155/2016/7806438)

**Supplemental Figure 1**


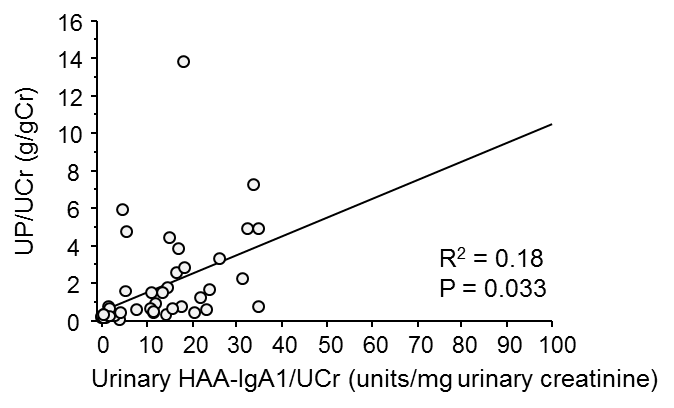

Supplement: Supplementary file 1 — Supplemental Figure 1. In renal-disease-control subjects, the correlation coefficient between the levels of urinary Gd-IgA1 and proteinuria was low (R2=0.180, P=0.033) compared to that in patients with IgAN (see Figure 4 for comparison). [file 7806438.f1.docx]
